# A Pharmacovigilance Approach for Post-Marketing in Japan Using the Japanese Adverse Drug Event Report (JADER) Database and Association Analysis

**DOI:** 10.1371/journal.pone.0154425

**Published:** 2016-04-27

**Authors:** Masakazu Fujiwara, Yohei Kawasaki, Hiroshi Yamada

**Affiliations:** 1 Department of Drug Evaluation & Informatics, Graduate school of Pharmaceutical Sciences, University of Shizuoka, Shizuoka, Japan; 2 Department of Biostatistics, Shionogi Pharmaceutical Company, Osaka, Japan; Kyushu University, JAPAN

## Abstract

**Background:**

Rapid dissemination of information regarding adverse drug reactions is a key aspect for improving pharmacovigilance. There is a possibility that unknown adverse drug reactions will become apparent through post-marketing administration. Currently, although there have been studies evaluating the relationships between a drug and adverse drug reactions using the JADER database which collects reported spontaneous adverse drug reactions, an efficient approach to assess the association between adverse drug reactions of drugs with the same indications as well as the influence of demographics (e.g. gender) has not been proposed.

**Methods and Findings:**

We utilized the REAC and DEMO tables from the May 2015 version of JADER for patients taking antidepressant drugs (SSRI, SNRI, and NaSSA). We evaluated the associations using association analyses with an apriori algorithm. Support, confidence, lift, and conviction were used as indicators for associations. The highest score in adverse drug reactions for SSRI was obtained for "aspartate aminotransferase increased", "alanine aminotransferase increased", with values of 0.0059, 0.93, 135.5, and 13.9 for support, confidence, lift and conviction, respectively. For SNRI, "international normalized ratio increased", "drug interaction" were observed with 0.0064, 1.00, 71.9, and NA. For NaSSA, "anxiety", "irritability" were observed with 0.0058, 0.80, 49.9, and 4.9. For female taking SSRI, the highest support scores were observed in "twenties", "suicide attempt", whereas "thirties", "neuroleptic malignant syndrome" were observed for male. Second, for SNRI, "eighties", "inappropriate antidiuretic hormone secretion" were observed for female, whereas "interstitial lung disease" and "hepatitis fulminant" were for male. Finally, for NaSSA, "suicidal ideation" was for female, and "rhabdomyolysis" was for male.

**Conclusions:**

Different combinations of adverse drug reactions were noted between the antidepressants. In addition, the reported adverse drug reactions differed by gender. This approach using a large database for examining the associations can improve safety monitoring during the post-marketing phase.

## Introduction

In the United States and Japan, the decision to approve a drug for clinical use is based on its having a satisfactory balance of benefits and risks within the conditions specified in the package insert. Information regarding the benefits and risks of a drug is derived from clinical trials that have been conducted prior to approval. However, information relating to a drug’s safety profile can change over time as its use is expanded in terms of patient characteristics and the number of patients exposed. In particular, during the early post-marketing period, the product might be used in settings different from clinical trials and in much larger populations—increasing the potential number of patients exposed within a relatively short timeframe. As such, new information will be continually generated once a drug is marketed, which can have an impact on its benefits and risk profiles. Therefore, a detailed evaluation of the information generated through pharmacovigilance activities is important for all products to ensure their safe use. Pharmacovigilance practices can improve information feedback to medical care providers and their patients in a timely manner, thereby reducing the overall risk to patients. With these ideas at the forefront, the International Conference on Harmonization of Technical Requirements for Registration of Pharmaceuticals for Human Use (ICH) created the E2E guidelines for pharmacovigilance planning [1].

The ICH E2E guidelines include information about how to summarize the important risks identified for a drug, important potential risks, and important missing information, which may include potentially at-risk populations and situations where the drug is likely to be used that have not been studied before approval. Within the ICH E2E guidelines, the U.S. food and drug administration (FDA) issued guides for premarketing risk assessment, good pharmacovigilance practices, and pharmacoepidemiologic assessments [2–3].

In Japan, the “Risk Management Plan Guidance” [4], issued in 2012, describes the basic ideas needed to develop a drug risk management plan, including safety considerations, a drug safety monitoring plan, and a risk minimization plan based on the ICH E2E guidelines. Safety considerations listed in the Japanese “Risk Management Plan Guidance” include important identified risks, important potential risks, and important missing information. Important potential risks include important adverse events that have been associated with a drug, but that have not been confirmed from clinical trials. Specifically, these may include adverse drug reactions that have been observed in drugs with the same indications, but not observed in a specific drug. Evaluation of information about adverse drug reactions observed for drugs with the same indications is possible through the drug package inserts.

In addition to package inserts, information about adverse drug reactions observed in drugs with the same indications can be acquired through the Japanese Adverse Drug Event Report (JADER) database. Here, adverse drug reactions are reported and managed by the Pharmaceuticals and Medical Devices Agency (PMDA) during the post-marketing phase. Studies investigating possible associations between a drug and adverse drug reactions have been proposed in viewpoint of signal detections using JADER [5–7]. In Japan, the Japan Pharmaceutical Information Center (JAPIC), which provides information on adverse drug reactions during the post-marketing phase, has been utilized in studies investigating drug-drug interactions and drug-target interactions [8–10]. The FDA Adverse Event Reporting System (FAERS) has features similar to those of JADER and has also been used for studies assessing associations between drugs and adverse drug reactions, drug-drug interactions, and drug-target interactions [11–13]. However, for adverse drug reactions occurring in drugs with the same indications, the data assessing the associated adverse drug reactions and the influence of demographics are insufficient.

In this paper, we consider the situation in which a new drug has been approved and has been in preparation for marketing. Under such circumstances, we propose using JADER as an evaluation tool for determining the association among adverse drug reactions that occur in drugs with the same indications along with the association between background information and adverse drug reactions. In order to evaluate potential associations, we used a data mining method applied to the JADER database. This approach could be an efficient evaluation method for identifying important potential risks as outlined in the Japanese “Risk Management Plan Guidance” and is expected to lead to higher quality security information monitoring.

For this study, we focused on antidepressant drugs. Various cohort studies have been performed during the post-marketing phase of antidepressant drugs in order to evaluate safety. For example, Jick et al. (2004) investigated the relationship between antidepressant use and suicide [14]. Weeke et al. (2012) investigated the association between antidepressant use and out-of-hospital cardiopulmonary arrest [15]. Because the safety profiles for antidepressants have been extensively evaluated after being commercially available, there is a great deal of additional safety information about new antidepressant drugs before arriving on the market. Here, we examine related adverse events and the influence of demographics within the Selective Serotonin Reuptake Inhibitors (SSRI), Serotonin and Norepinephrine Reuptake Inhibitors (SNRI), and Noradrenergic and Specific Serotonergic Antidepressant (NaSSA) antidepressant drug categories. We targeted seven drugs: fluvoxamine, paroxetine, sertraline, escitalopram, milnacipran, duloxetine, and mirtazapine.

## Methods

The JADER dataset was downloaded from PMDA’s homepage (http://www.pmda.go.jp/) and included four tables as follows: 1) DEMO table (gender, age, weight, etc.), 2) drug table (drug name, causality of drug (suspected drug or concomitant drug), etc.), 3) REAC table (adverse drug reaction name, outcomes, etc.), and 4) MH table (medical history names, etc.). We used the REAC and DEMO table data taken from the JADER database updated in May 2015 for patients taking SSRI, SNRI and NaSSA antidepressants.

We evaluated the associations between adverse drug reactions and demographics using an apriori algorithm [16–18]. Support, confidence, lift, and conviction were used as indicators to assess the association of X and Y (X and Y being adverse drug reactions or demographic information) [19]. An apriori algorithm is a data mining method for extracting frequent combinations from a large database that can efficiently find sets of adverse drug reactions that occur more frequently than the minimum support threshold (defined as 0.001 in this study). After that, it generates sets of adverse drug reactions with the minimum confidence threshold (defined as 0.8 in this study).

**Fig 1:**
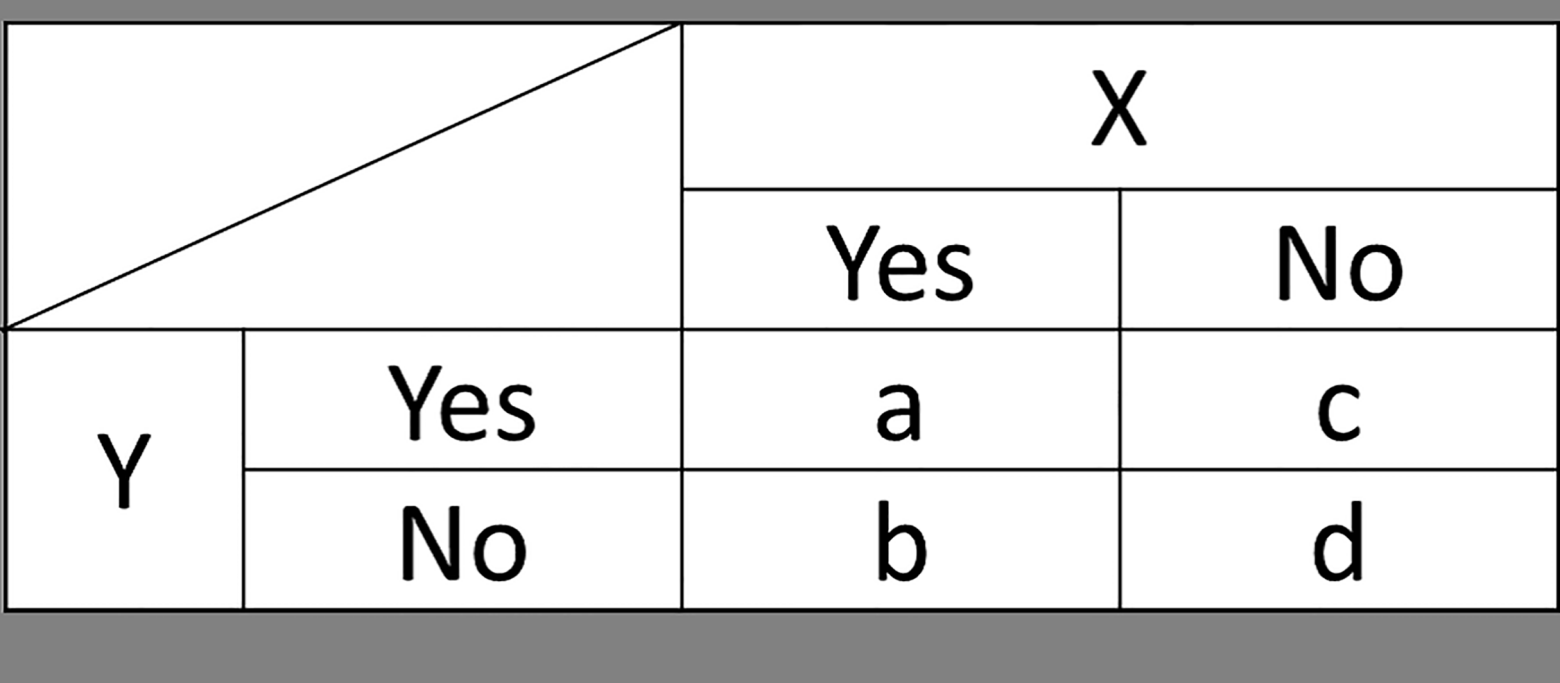
Assessment of associations based on the presence or absence of adverse drug reactions and/or demographics. (i.e., a = number of patients who reported the adverse drug reactions X and Y; b = number of patients who reported X, but not Y; c = number of patients who did not report X, but reported Y; and d = number of patients who did not report X or Y).

Patients who experienced adverse drug reactions (more than one adverse drug reactions) were denoted by "Yes," whereas patients who did not experience adverse drug reactions (more than one adverse drug reactions) were denoted as "No"; in this way, we developed a contingency table for adverse drug reactions, X and Y, as shown in Fig 1.

When considering the association among adverse drug reactions, patients were stratified according to whether or not they experienced specific adverse drug reactions, i.e. "a" in Fig 1 reflects the number of patients who have reported the adverse drug reaction X and the adverse drug reaction Y. In addition, the total number of reported patients is expressed as “t.” The equations for determining associations between X and Y are defined as follows:

Support=a/t

Confidence=a/(a+b)

Lift={a/(a+b)}/{(a+c)/t}

Conviction={(c+d)/t}/{b/(a+b)}

Two association analyses were performed. First, we evaluated the associations between adverse drug reactions using the REAC table reported for the SSRI, SNRI, and NaSSA, assigning adverse drug reactions to the X and Y variables. Second, we evaluated the associations between gender and adverse drug reactions by using the REAC table and the DEMO table. Specifically, we assessed the association between gender and adverse drug reactions by assigning gender to the Y variable and adverse drug reactions to the X variable as reported for the SSRI, SNRI, NaSSA, and demographic information (age category). We performed these analyses using the apriori function of arules library in the arules package of R version 3.1.2 software [20].

## Results

### Associations between adverse drug reactions from SSRI, SNRI, and NaSSA antidepressants

In the JADER database, which was updated in May 2015, there were 345,715 cases reported for adverse drug reactions, and within these cases the total number of adverse drug reactions was 549,508. In addition, there were 4377, 935, and 686 cases of reported adverse drug reactions for SSRI, SNRI, and NaSSA, respectively. Within the reported cases, there were a total of 8462, 1417, and 1084 adverse drug reactions for SSRI, SNRI, and NaSSA, respectively.

The association analyses were applied to the adverse drug reactions data for SSRI, SNRI, and NaSSA, respectively. Support, confidence, lift, and conviction for each association rule are shown in Table 1; the association rules up to fifth in the descending order of the support are shown.

**Table 1:** Association Rules for the Adverse Drug Reactions Reported for SSRI, SNRI, and NaSSA.

Type	X	Y	Support	Confidence	Lift	Conviction
SSRI	Aspartate aminotransferase increased	Alanine aminotransferase increased	0.0059	0.93	135.5	13.9
	Alanine aminotransferase increased	Alanine aminotransferase increased	0.0059	0.87	135.5	7.5
	Gamma-glutamyl transferase increased, Aspartate aminotransferase increased	Alanine aminotransferase increased	0.0032	1.00	145.9	NA
	Gamma-glutamyltransferase increased, Aspartate aminotransferase increased	Aspartate aminotransferase increased	0.0032	0.88	136.8	7.9
	Blood lactate dehydrogenase increased	Alanine aminotransferase increased	0.0021	0.82	119.4	5.5
SNRI	International normalised ratio increased	Drug interaction	0.0064	1.00	71.9	NA
	Irritability	Anxiety	0.0064	0.86	114.5	6.9
	Anxiety	Irritability	0.0064	0.86	114.5	6.9
	Aspartate aminotransferase increased	Alanine aminotransferase increased	0.0032	1.00	233.8	NA
	Cardio-respiratory arrest	Pulmonary oedema	0.0032	1.00	311.7	NA
NaSSA	Anxiety	Irritability	0.0058	0.80	49.9	4.9
	Multi-organ failure	Agranulocytosis	0.0044	1.00	98.0	NA
	Confusional state	Hallucination, visual	0.0029	1.00	57.2	NA
	Hypothyroidism	Myopathy	0.0029	1.00	228.7	NA
	Cerebellar ataxia	Altered state of consciousness	0.0029	1.00	52.8	NA

The association rules for SSRI with the highest score for support were X = aspartate aminotransferase increased and Y = alanine aminotransferase increased. The values for support, confidence, lift, and conviction were 0.0059, 0.93, 135.5, and 13.9, respectively. The association rules for SNRI with the highest score for support were X = international normalized ratio increased and Y = drug interaction. The values for support, confidence, lift, and conviction were 0.0064, 1.00, 71.9, and NA, respectively; values denotes as NA had a zero denominator in the formula for the conviction and therefore could not be calculated. The association rules for NaSSA with the highest score for support were X = anxiety and Y = irritability. The values for support, confidence, lift, and conviction were 0.0058, 0.80, 49.9, and 4.9, respectively.

### Associations between adverse drug reactions and demographics

In JADER, the reported number of adverse drug reaction cases for women was 2562, 509, and 392 for SSRI, SNRI, and NaSSA antidepressants, respectively. Within these reported cases, the total number of adverse drug reactions was 4946, 765, and 618 for SSRI, SNRI, and NaSSA, respectively. For men, the reported number of adverse drug reaction cases was 1678, 418, and 286 for SSRI, SNRI, and NaSSA, respectively. Within these reported cases, the total number of adverse drug reactions was 3206, 642, and 452 for SSRI, SNRI, and NaSSA, respectively. Association analyses were also performed using background information (categorized as age), where the adverse drug reactions for SSRI, SNRI, and NaSSA were set as X, and sex (male or female) was set as Y. Support, confidence, lift and conviction for each association rule are shown in Table 2. The association rules up to third in the descending order of support by gender are shown. The association between SSRI and female (Y) with the highest score for support was X = twenties and suicide attempt. In contrast, the association between SSRI and male (Y) with the highest score for support was X = thirties and neuroleptic malignant syndrome.

**Table 2:** Association Rules for the Adverse Drug Reactions Reported for SSRI, SNRI, and NaSSA.

Type	X	Y	Support	Confidence	Lift	Conviction
SSRI	Twenties, Suicide attempt	Female	0.012	0.81	1.39	2.21
	Seventies, Serotonin syndrome	Female	0.0087	0.83	1.41	2.38
	Self injurious behaviour	Female	0.0059	0.81	1.39	2.21
	Thirties, Neuroleptic malignant syndrome	Male	0.0046	0.91	2.37	6.78
	Forties, Aggression	Male	0.0027	1.00	2.61	NA
	Respiratory disorder	Male	0.0023	0.83	2.17	3.70
SNRI	Eighties, Inappropriate antidiuretic hormone secretion	Female	0.02	0.87	1.60	3.49
	Twenties, Drug eruption	Female	0.011	1.00	1.84	NA
	Sixties, Inappropriate antidiuretic hormone secretion	Female	0.011	0.91	1.67	5.01
	Interstitial lung disease	Male	0.0096	0.90	2.01	5.53
	Hepatitis fulminant	Male	0.0096	0.90	2.01	5.53
	Thirties, Neuroleptic malignant syndrome	Male	0.0086	1.00	2.24	NA
NaSSA	Suicidal ideation	Female	0.035	0.89	1.56	3.86
	Akathisia	Female	0.019	0.81	1.42	2.29
	Inappropriate antidiuretic hormone secretion	Female	0.015	0.83	1.46	2.57
	Rhabdomyolysis	Male	0.017	0.80	1.92	2.92
	Thirties, Rhabdomyolysis	Male	0.0087	0.86	2.06	4.08
	Dystonia	Male	0.0073	1.00	2.40	NA

The association between SNRI and female (Y) with the highest score for support was X = eighties and inappropriate antidiuretic hormone secretion; for male patients, the highest score for support was X = interstitial lung disease. Finally, the association between NaSSA and female (Y) with the highest score for support was X = suicidal ideation; for male patients, the highest score for support was X = rhabdomyolysis. For all drugs examined (SSRI, SNRI, and NaSSA), support for female patients had higher values compared to male patients.

## Discussion

In this study, we propose a new approach for improving safety monitoring of drugs that have been recently approved and are moving toward marketing that evaluates the associations between adverse drug reactions reported for drugs with the same indications, as well as the associations between demographics and adverse drug reactions by utilizing the JADER database. When new antidepressants are developed and approved by regulatory agencies, the approach described should be conducted, after which the results obtained using the approach should be shared with medical representatives so that doctors can be alerted to take precautions against combinations of adverse drug reactions. Our new method should improve drug safety monitoring, because current safety monitoring approaches focus only on individual adverse drug reactions.

Support values from two analyses of antidepressants (SSRI, SNRI, and NaSSA) were small; therefore, the rate of each association rule was considered to be small. However, confidence values, which are taken from zero to one with a higher value denoting a stronger association between X and Y, were close to one. In addition, lift and conviction values were greater than one in nearly all instances—suggesting a strong association. These data suggest that each association considered was strong, although the expression rates were low. Furthermore, most of the adverse drug reactions considered in this study had previously been documented as known risks for antidepressant drugs [21, 22].

Although the specific adverse drug reaction combinations listed in Tables 1 and 2 differ for SSRI, SNRI, and NaSSA, we considered them to be specific adverse drug reaction combinations for SSRI, SNRI, and NaSSA. Understanding the associations among adverse drug reactions that may occur for antidepressants or other drugs with similar indications will enable us to do high-quality safety monitoring.

Gender considerations are important, as the association values for women were generally higher compared with the values for men. It is known that the number of women patients with depression is higher than for men [23]; this was confirmed from these results.

From Table 2, the associated adverse drug reactions and age information differed by gender, suggesting that safety information monitoring by gender should be performed. This approach, using association analyses from data within the JADER database is beneficial and considers not only the frequency of adverse drug reactions, but also combinations among adverse drug reactions. One limitation of this study is the spontaneous nature of the adverse event reporting within the JADER database; adverse drug reactions may be reported more frequently and may include reporting bias. Therefore, the results of the association analyses should be interpreted in consideration of clinical perspectives.

This approach, using association analyses, can also be used in the development stage of new drugs. Specifically, by sharing analysis results with clinical research associates, it is possible to conduct clinical trials in consideration of safety of patients by further understanding the associations among adverse drug reactions of drugs with the same indications. Finally, this approach can also be applied to the FAERS. For drugs that will be marketed globally, evaluation of adverse drug reactions using FAERS and association analyses, including additional information (region, etc.) could lead to global monitoring based on safety information.
